# Supplementary material for: Astragalus polysaccharides and astragaloside IV alleviate inflammation in bovine mammary epithelial cells by regulating Wnt/β-catenin signaling pathway
Source: PLoS One. 2022 Jul 25;17(7):e0271598. doi: 10.1371/journal.pone.0271598 (PMC9312414; doi:10.1371/journal.pone.0271598)
Supplement: S1 Raw images — (PDF) [file pone.0271598.s001.pdf]

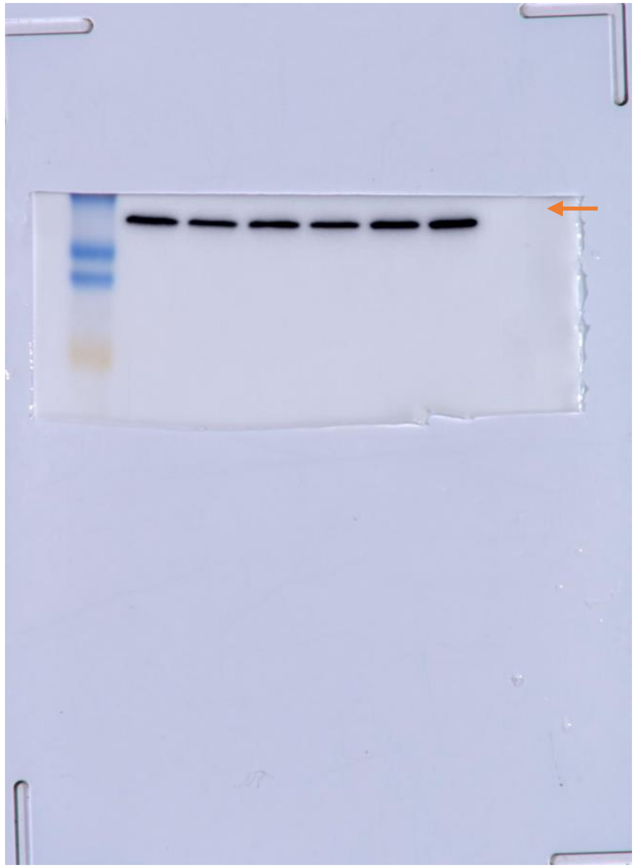

Fig1b β-actin

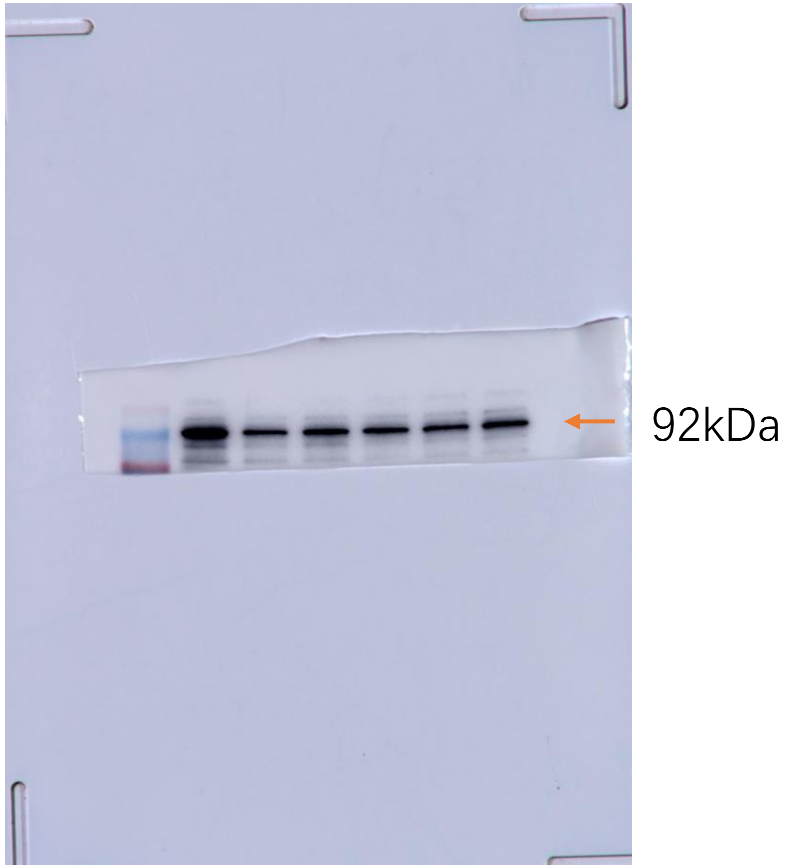

Fig1b β-catenin

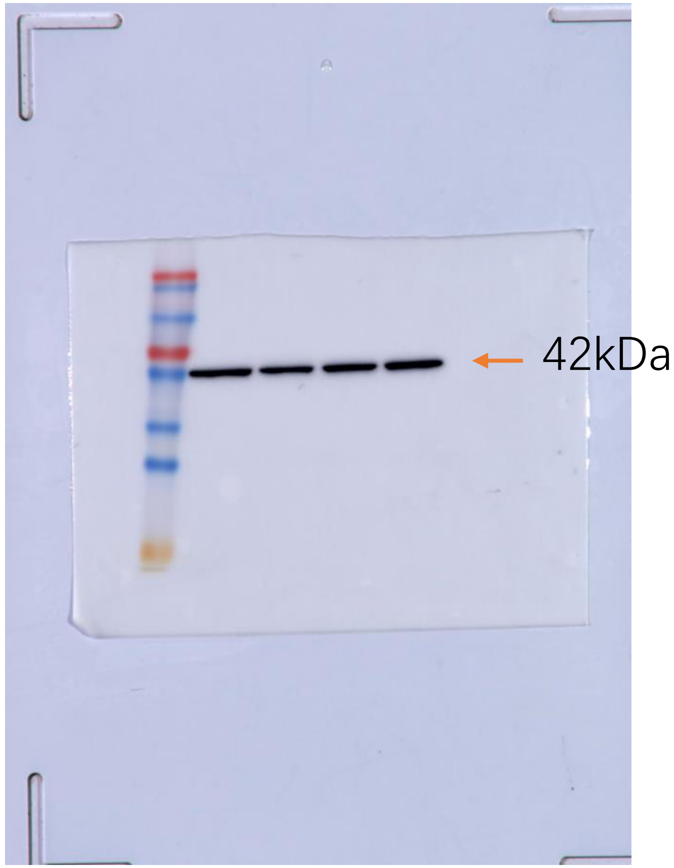

Fig3 β-actin

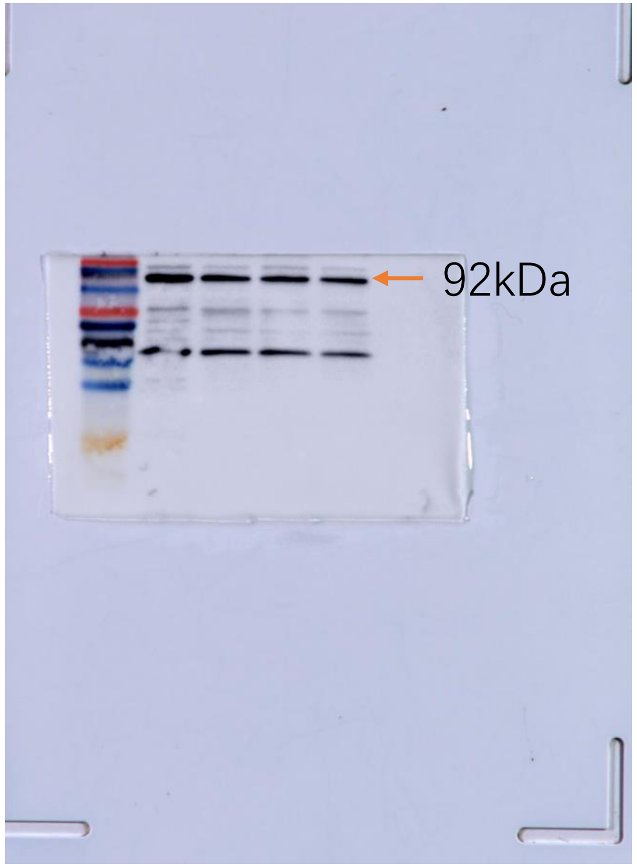

Fig3 β-catenin

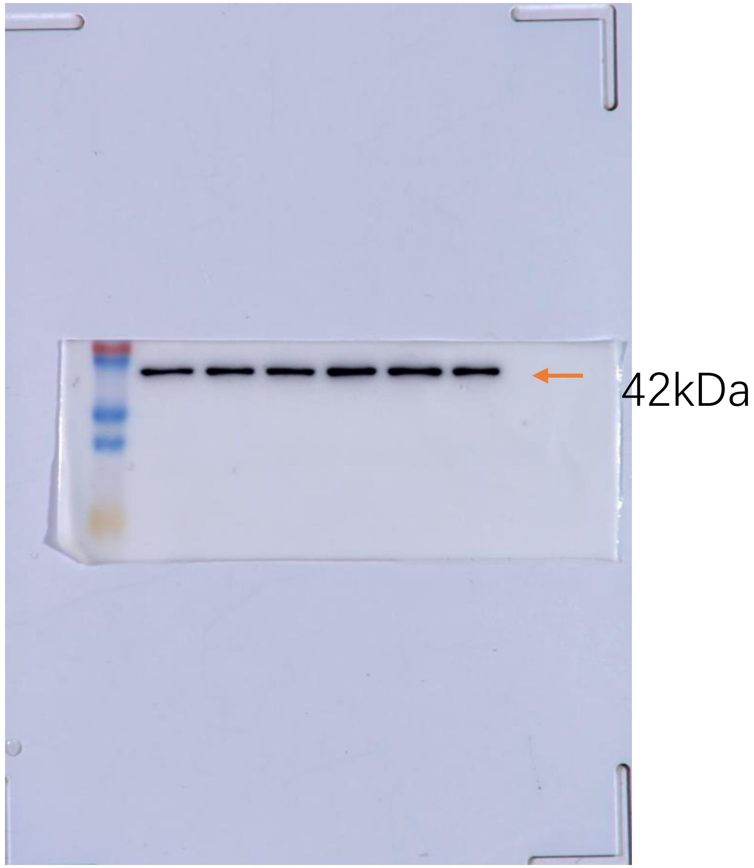

Fig4a β-actin

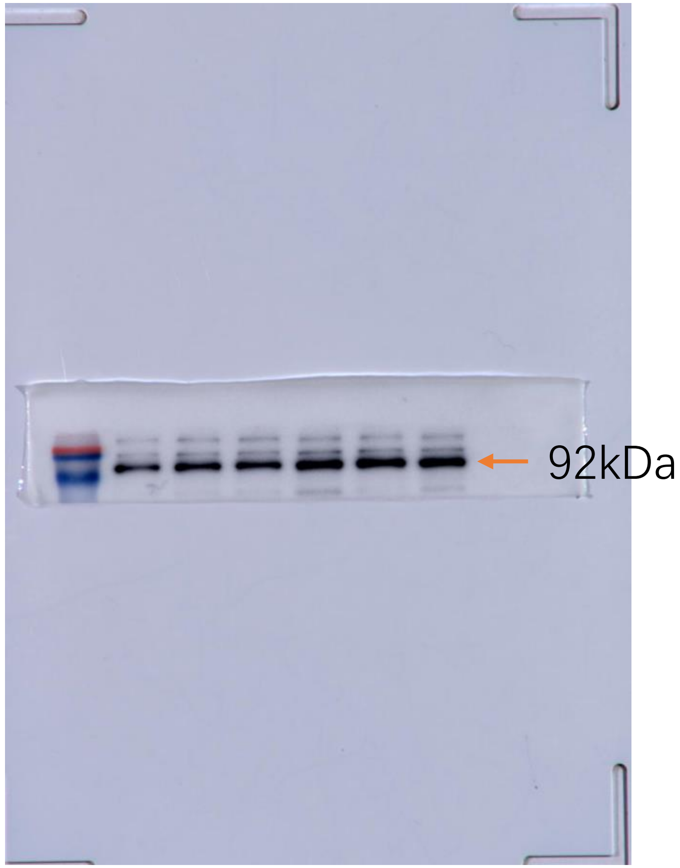

Fig4a β-catenin

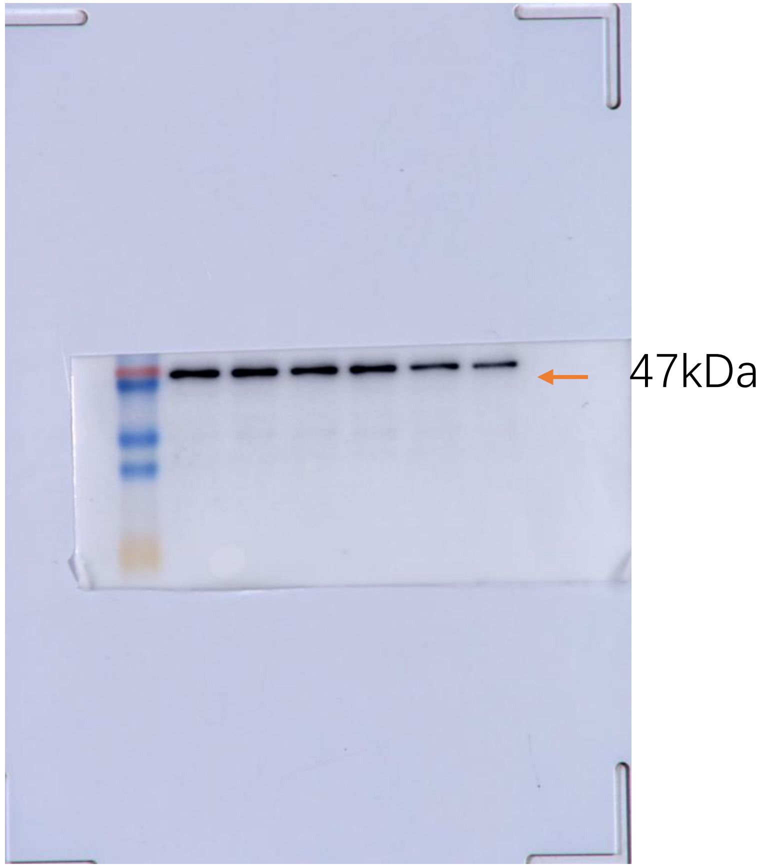

Fig4a phospho-GSK3β

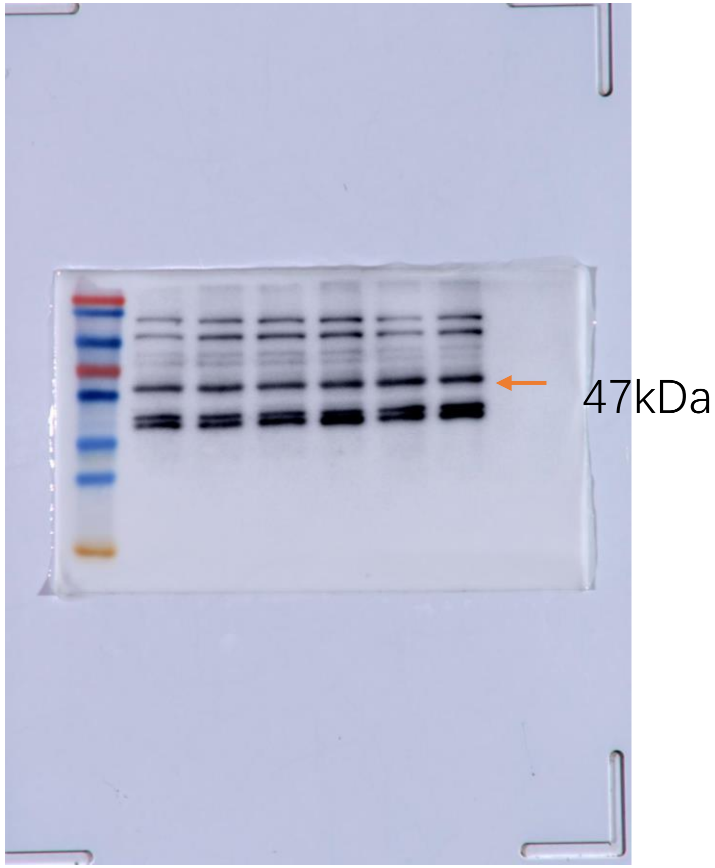

Fig4a GSK3β

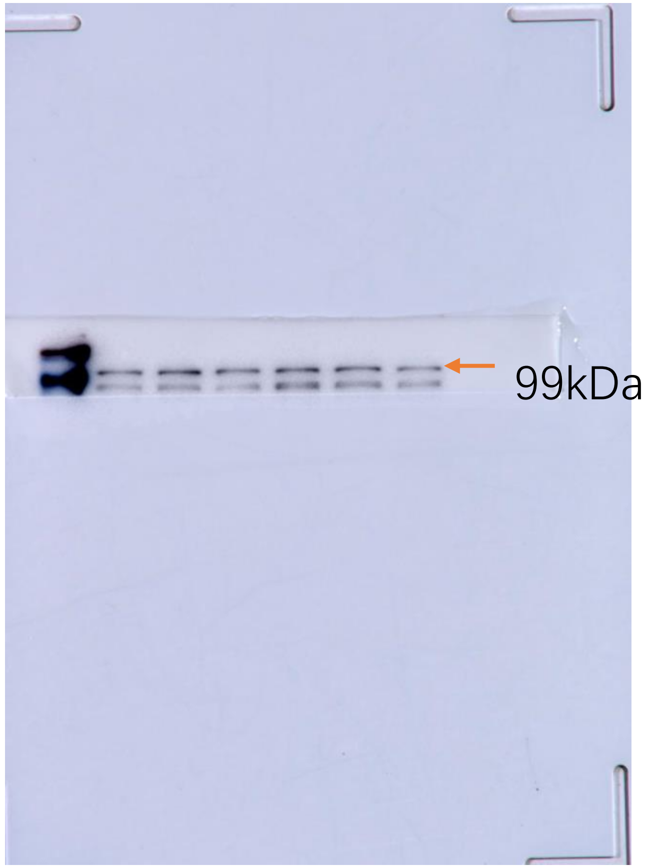

Fig4a AXIN1

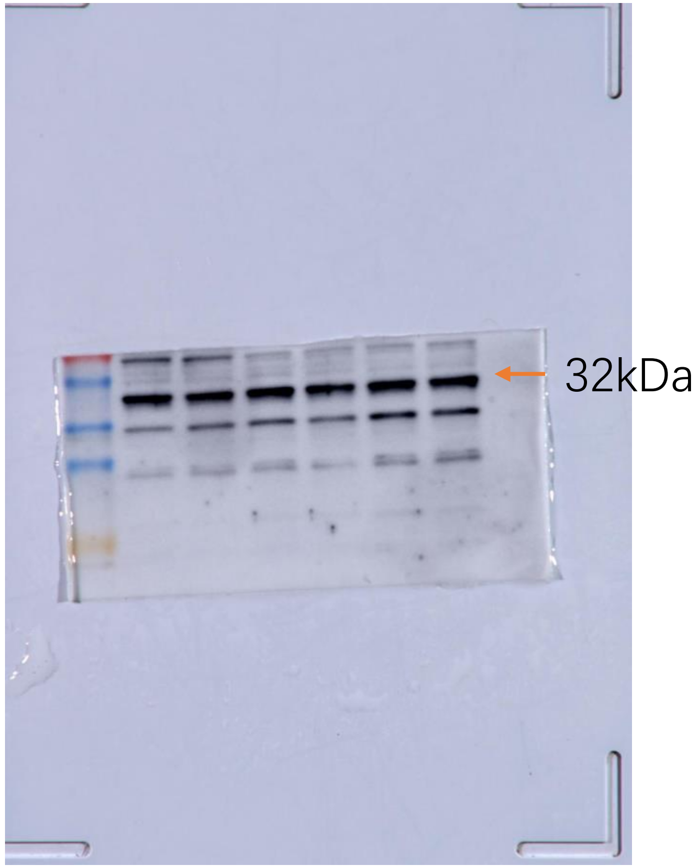

Fig4a cyclinD1

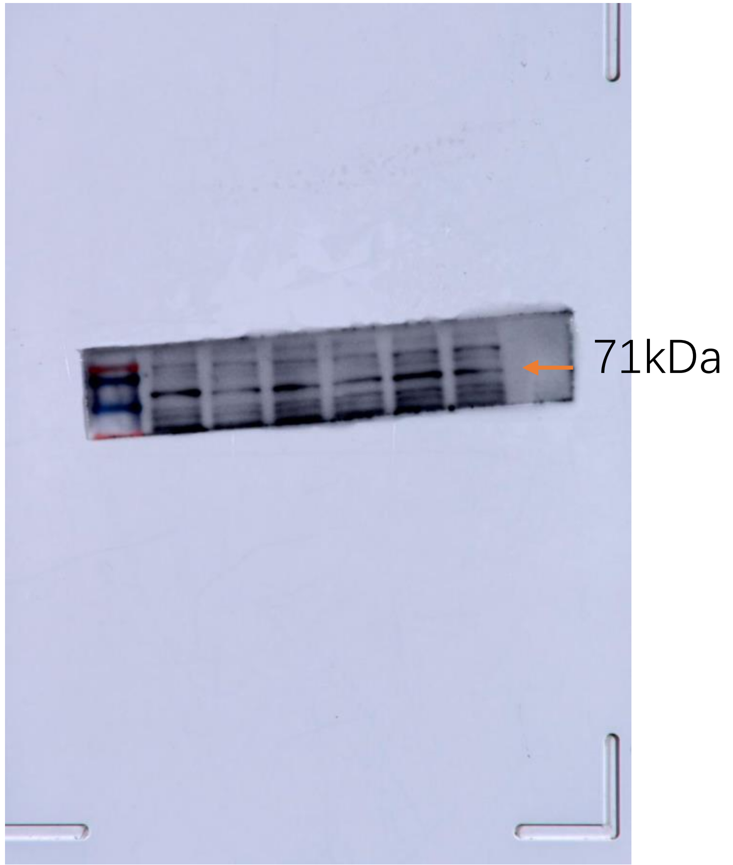

Fig4a TCF4

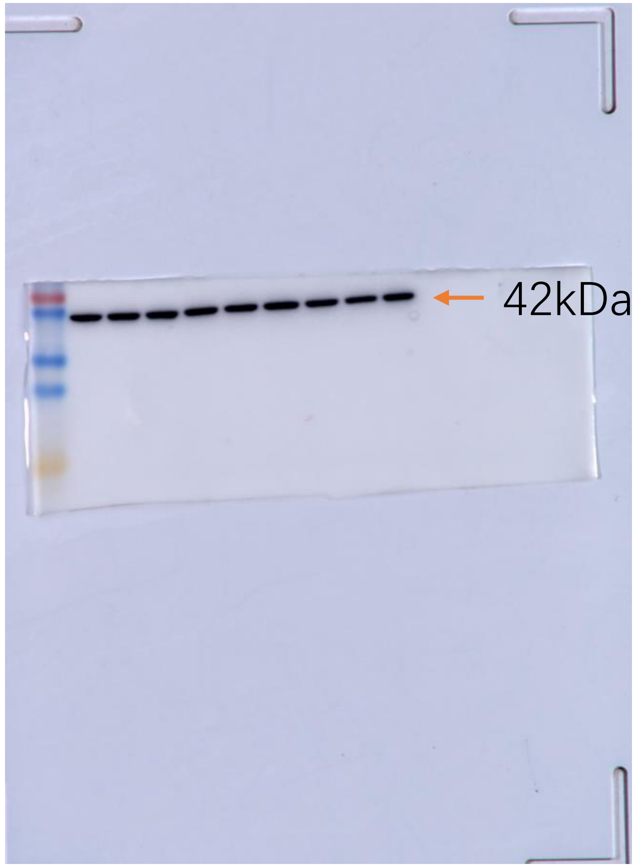

Fig4b β-actin

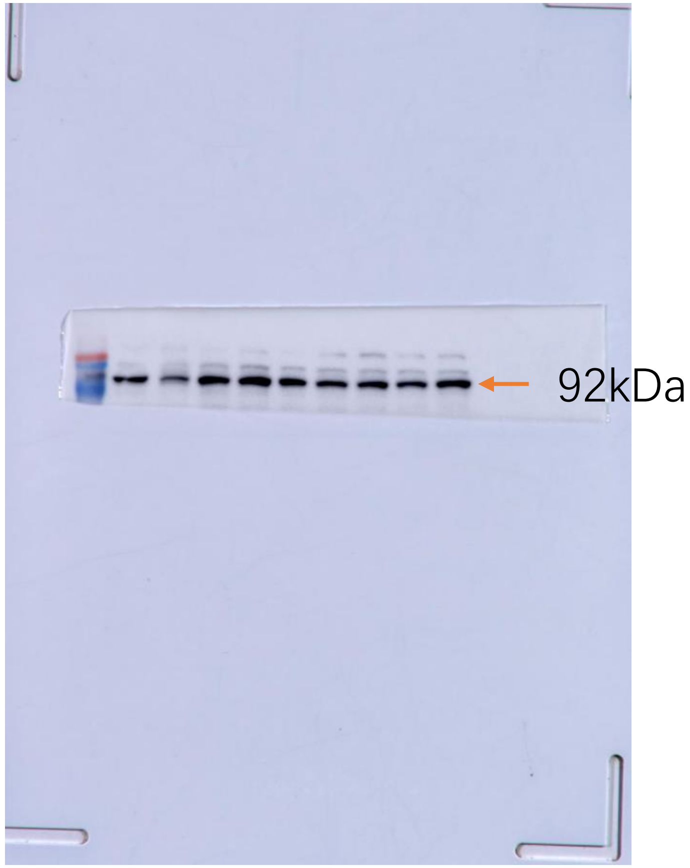

Fig4b β-catenin

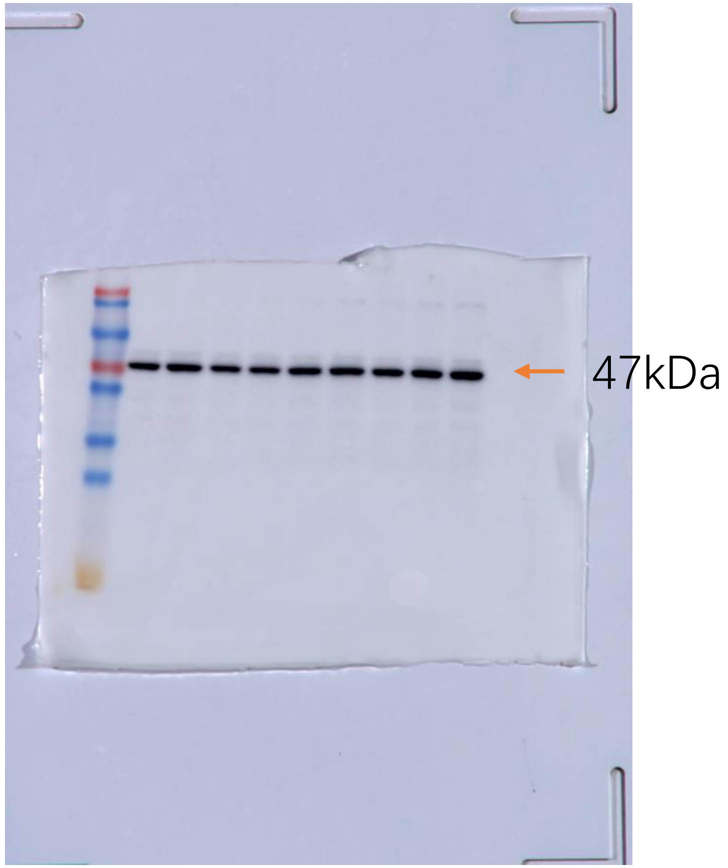

Fig4b phospho-GSK3β

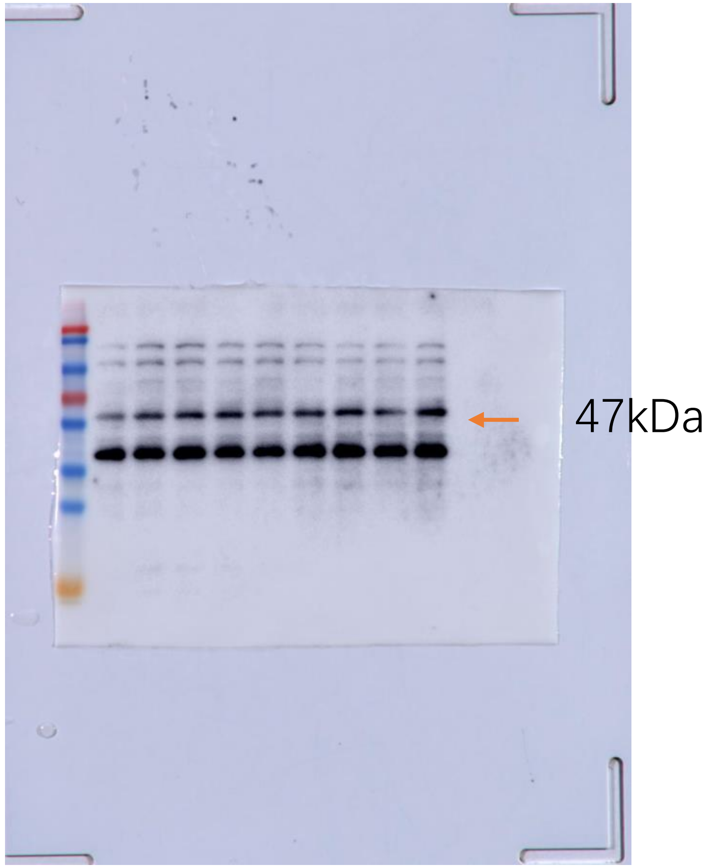

Fig4b GSK3β

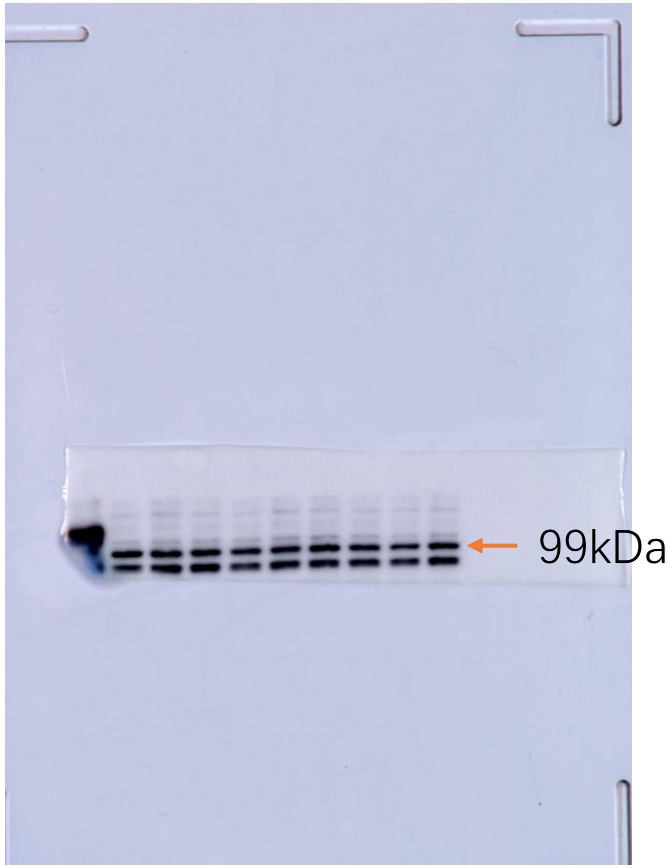

Fig4b AXIN1

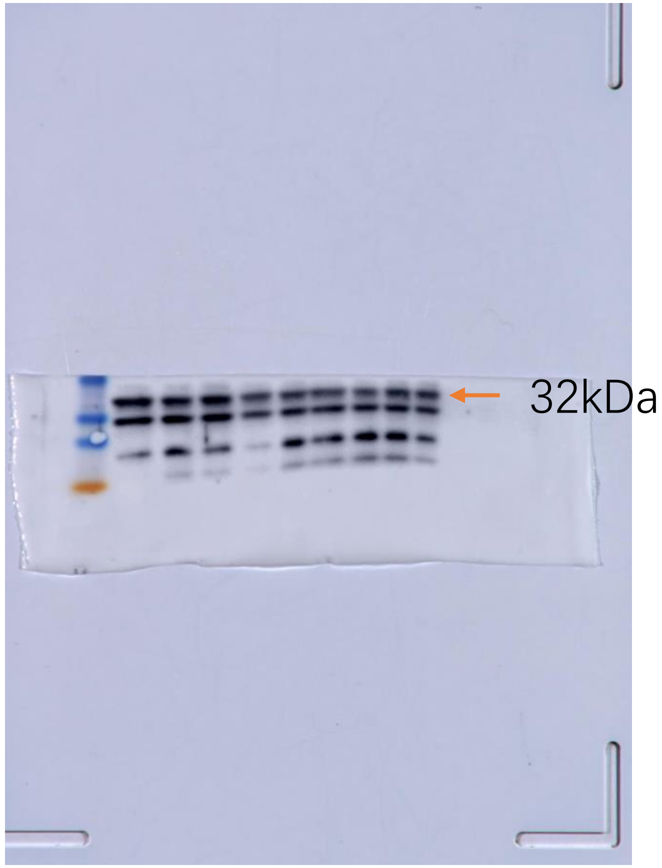

Fig4b cyclinD1

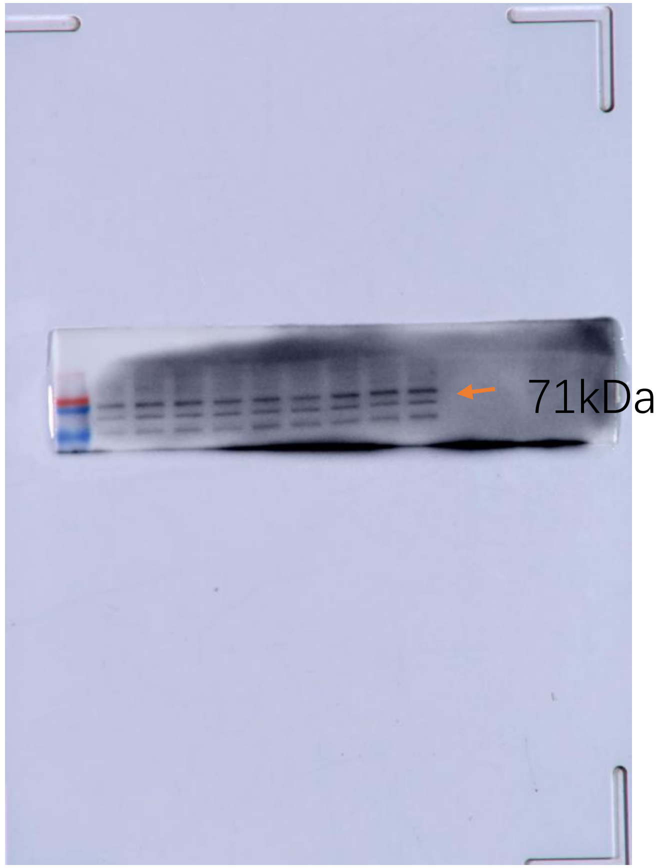

Fig4b TCF4

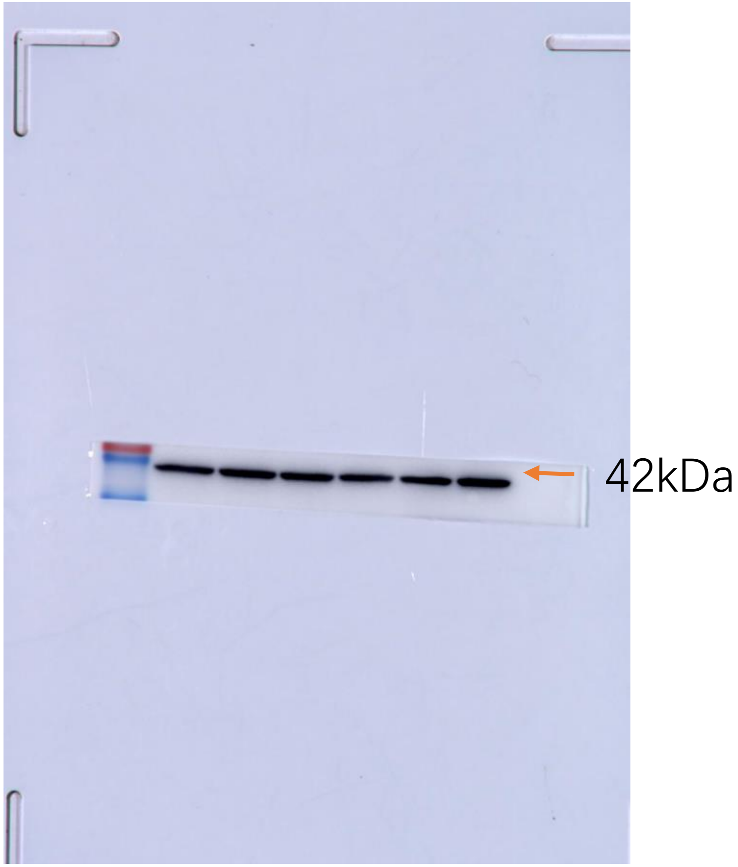

Fig4c β-actin

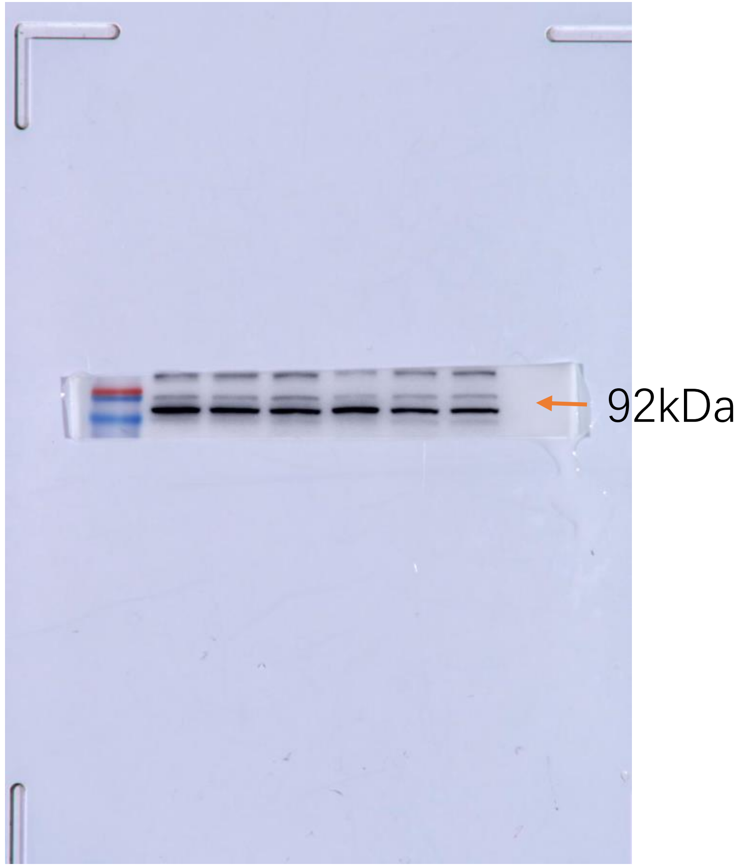

Fig4c β-catenin

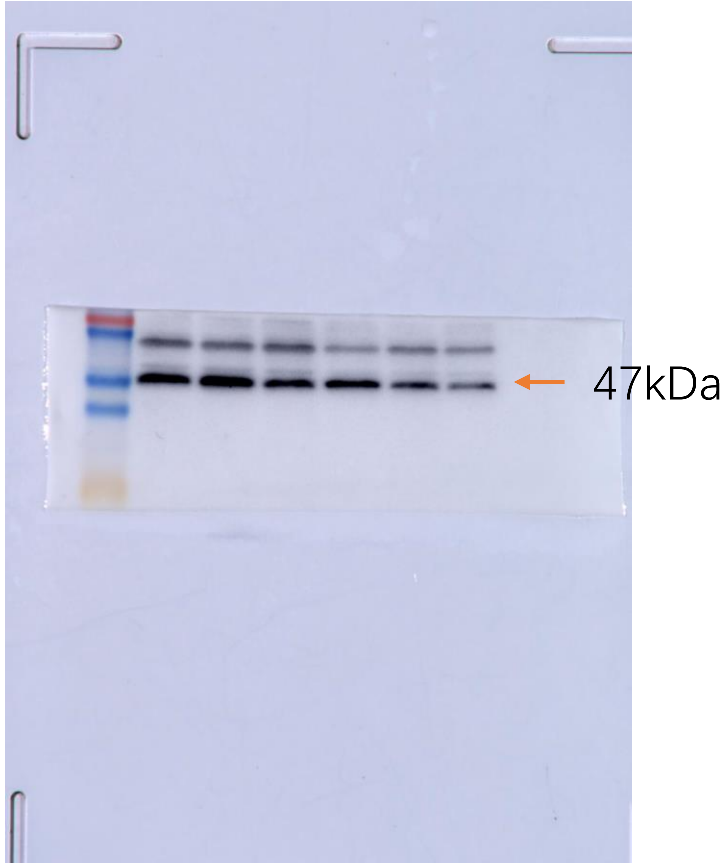

Fig4c phospho-GSK3β

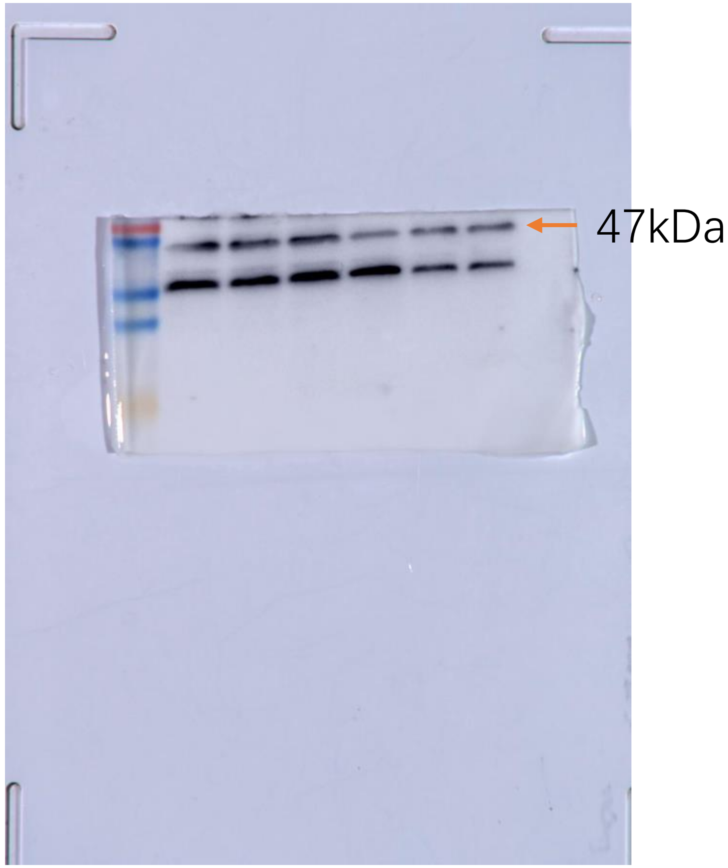

Fig4c GSK3β

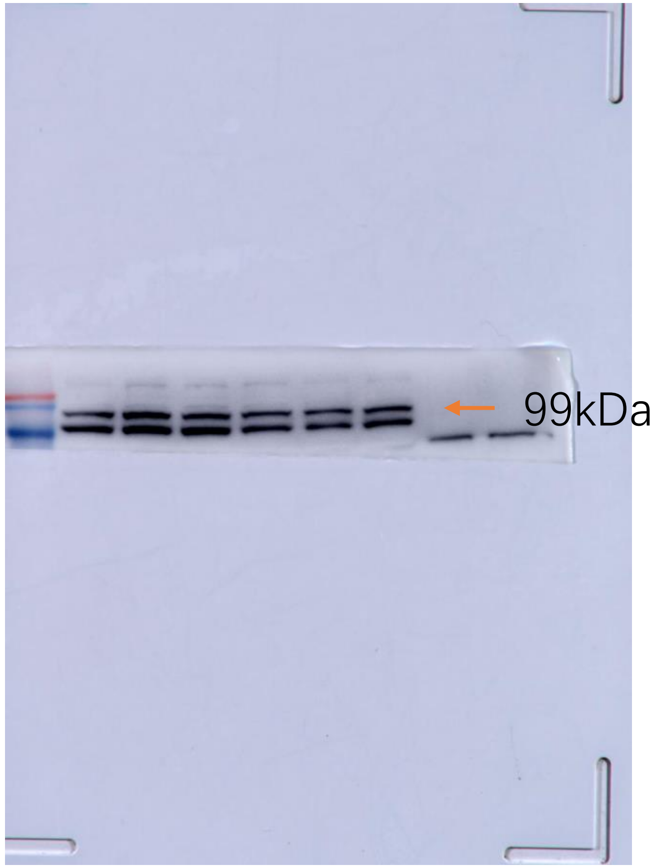

Fig4c AXIN1

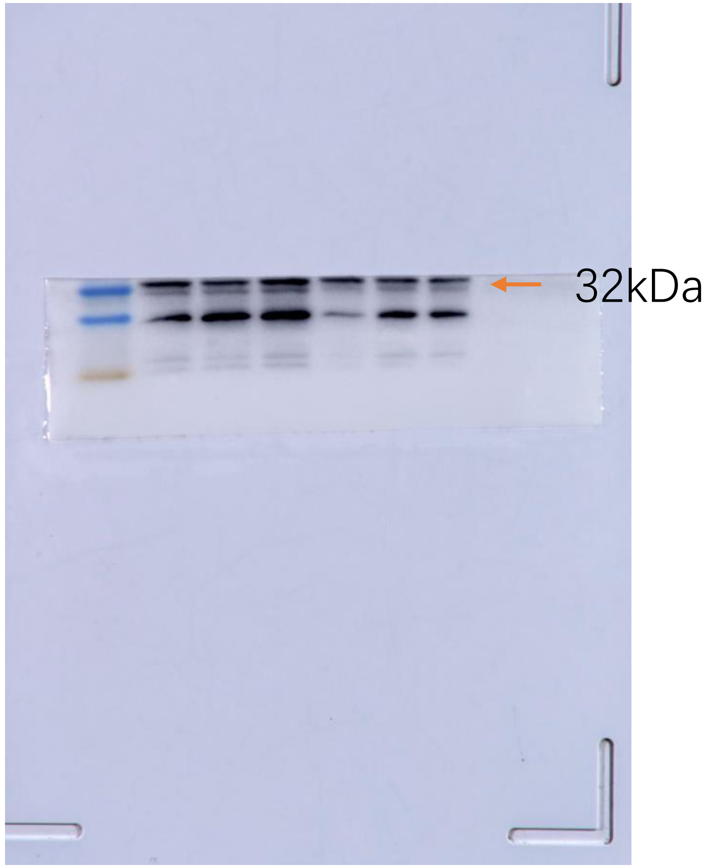

Fig4c cyclinD1

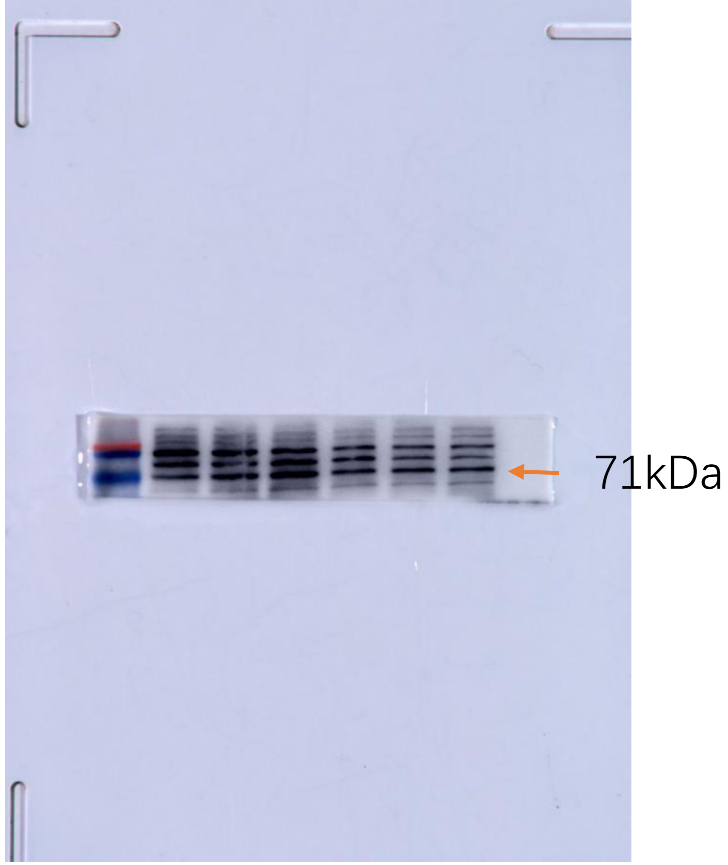

Fig4c TCF4

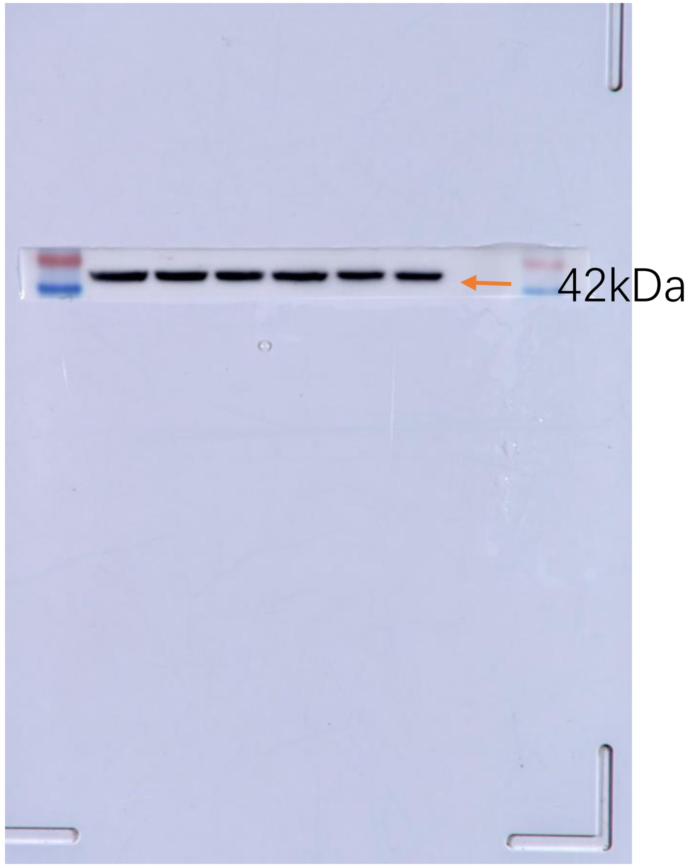

Fig4d β-actin

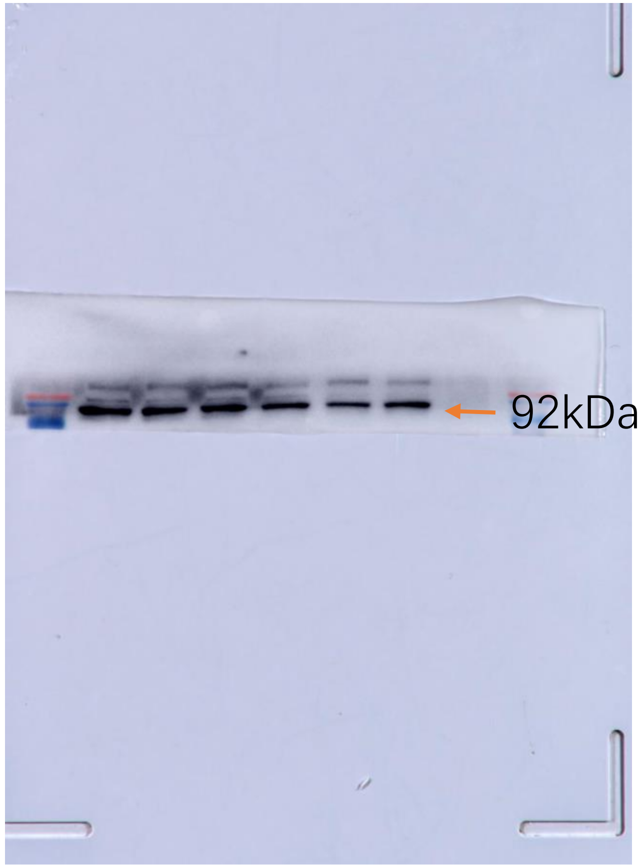

Fig4d β-catenin

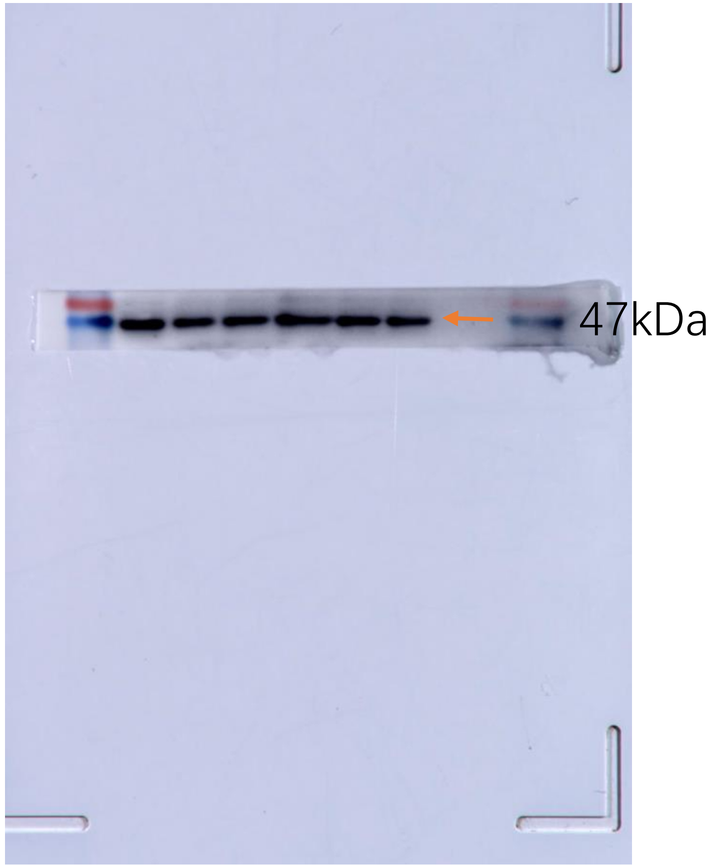

Fig4d GSK3β

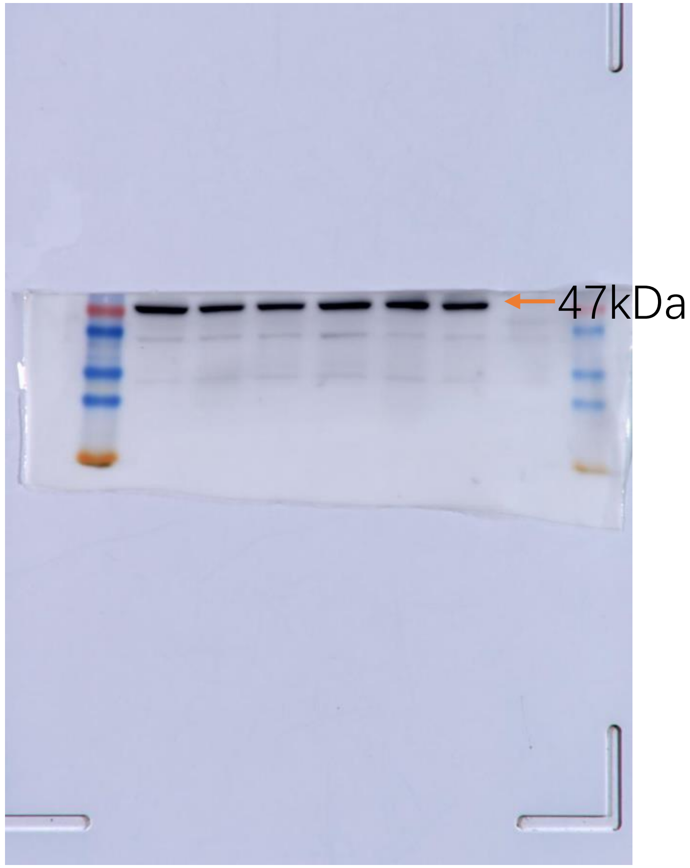

Fig4d phospho-GSK3β

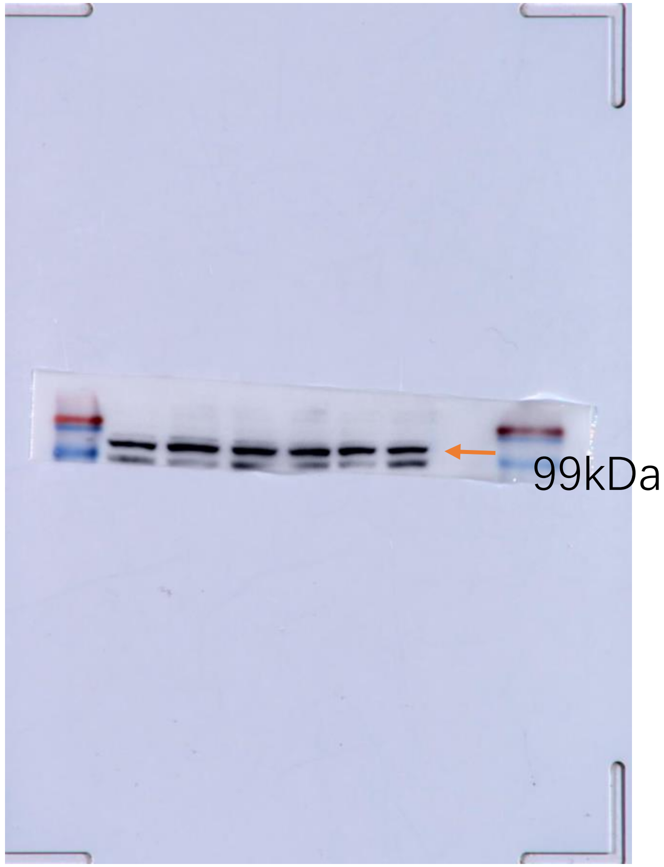

Fig4d AXIN1

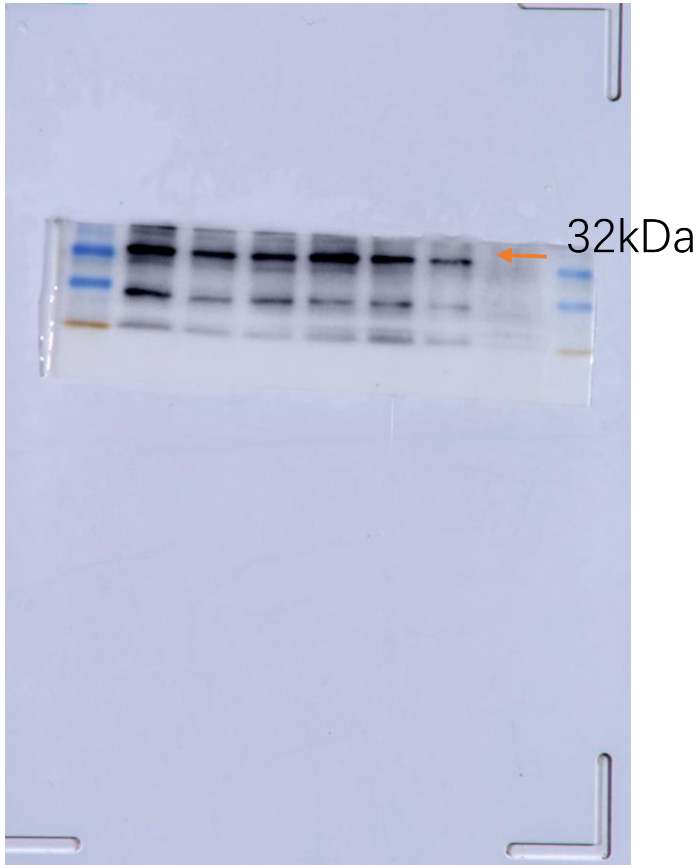

Fig4d cyclinD1

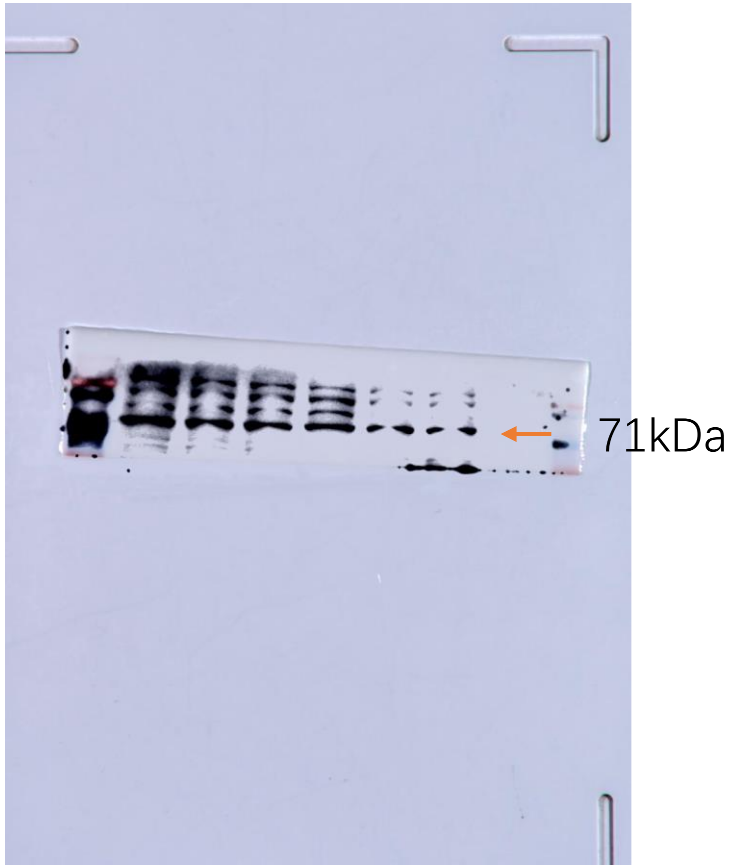

Fig4d TCF4
